# Differentiation of regions with atypical oligonucleotide composition in bacterial genomes

**DOI:** 10.1186/1471-2105-6-251

**Published:** 2005-10-14

**Authors:** Oleg N Reva, Burkhard Tümmler

**Affiliations:** 1 Klinische Forschergruppe, OE6711, Medizinische Hochschule Hannover, Carl-Neuberg-Strasse 1, D-30625 Hannover, Germany; 2 Danylo Zabolotny Institute of Microbiology and Virology of the National Academy of Science of Ukraine, Dep. of Antibiotics, 154 Zabolotnogo Str., D03680, Kyiv GSP, Ukraine

## Abstract

**Background:**

Complete sequencing of bacterial genomes has become a common technique of present day microbiology. Thereafter, data mining in the complete sequence is an essential step. New in silico methods are needed that rapidly identify the major features of genome organization and facilitate the prediction of the functional class of ORFs. We tested the usefulness of local oligonucleotide usage (OU) patterns to recognize and differentiate types of atypical oligonucleotide composition in DNA sequences of bacterial genomes.

**Results:**

A total of 163 bacterial genomes of eubacteria and archaea published in the NCBI database were analyzed. Local OU patterns exhibit substantial intrachromosomal variation in bacteria. Loci with alternative OU patterns were parts of horizontally acquired gene islands or ancient regions such as genes for ribosomal proteins and RNAs. OU statistical parameters, such as local pattern deviation (D), pattern skew (PS) and OU variance (OUV) enabled the detection and visualization of gene islands of different functional classes.

**Conclusion:**

A set of approaches has been designed for the statistical analysis of nucleotide sequences of bacterial genomes. These methods are useful for the visualization and differentiation of regions with atypical oligonucleotide composition prior to or accompanying gene annotation.

## Background

The number of sequenced prokaryotic genomes increases rapidly each year. Their comprehensive analysis requires the development of new high-throughput computational methods. The analysis of oligonucleotide usage biases has been recognized to be practical for the recognition of pathogenicity islands [1,2] and elucidation of origins of orphan sequences [3-5]. Recently we have developed methods for the global analysis of oligonucleotide usage (OU) in complete sequences of bacterial chromosomes, plasmids and phages [6]. The patterns of deviations of oligonucleotide frequencies from expectations were shown to be genome signatures reflecting to some extent the phylogenetic links between microorganisms [3,4,7,8].

The usage of oligonucleotides in bacterial sequences is not random. Frequencies of the oligonucleotide words (further – *words*) depend strongly on their physicochemical properties such as base stacking energy, propeller twist angle, bendability, position preference and protein deformability [6]. Oligonucleotide usage in bacterial genomes is strongly influenced by codon usage [9], however, there are further, yet unknown mechanisms of word selection [10].

To characterize OU in a sequence, the concept of OU patterns has been introduced [6]. Disparity of frequencies of words and their reverse complements termed as pattern skew (PS) and variance of oligonucleotide frequencies (OUV) are attributes of each OU pattern and the distance (D) expresses the difference between two OU patterns. These OU parameters are independent of the length of the sequence and hence allow the comparison of windows of different sequence length ([6] and see 'Materials and methods'). This study applied OU statistics to visualize and discern gene islands of different functional classes. The developed methods are of importance for structural, functional and comparative genomics.

## Results and discussion

### Types of OU patterns, abbreviations and nomenclature

Counts of words of different lengths *N *from 2 to 7-mer were analyzed in this work applying different schemes of normalization. Different types of OU patterns were abbreviated as *type*_*N-*mer. Types were "n0" for non-normalized, "n1" for normalized by mononucleotide frequencies, "n2" for normalized by dinucleotides and so on. For example, the non-normalized tetranucleotide usage pattern is denoted as n0_4 mer, trinucleotide usage pattern normalized by dinucleotides is n2_3 mer, pentanucleotide usage pattern normalized by trinucleotides is n3_5 mer. Each OU pattern is characterized by three statistical parameters: D – distance between two patterns of the same type (in this work we used distances D between local and global genome patterns); PS – pattern skew, distance between the two patterns of the direct and reverse strands of the same DNA sequence; and OUV – oligonucleotide usage variance. Correspondingly, the nomenclature is as follows: distance between a local n0_4 mer pattern and the corresponding global pattern – D:n0_4 mer; pattern skew of a n0_3 mer pattern – PS:n0_3 mer; variance of a n3_7 mer pattern – OUV:n3_7 mer. Two subtypes of normalization of local OU patterns were defined: normalized by frequencies of component words in the current genomic fragment (internal normalization, *i*) and in the complete sequence of the genome (global normalization, *g*). For example, internal and global OUV determined for a local n1_4 mer pattern were OUV:n1_*i*__4 mer and OUV:n1_*g*__4 mer, respectively. Internal normalization was always used in this study with the exception of the chapter "Identification of horizontally transferred elements" where the distances between OUV:n1_*i*__4 mer and OUV:n1_*g*__4 mer are analyzed. To simplify nomenclature, the index *i *was skipped in the pattern type abbreviation in all other chapters.

### OU constraints in bacterial DNA

**Figure 1:**
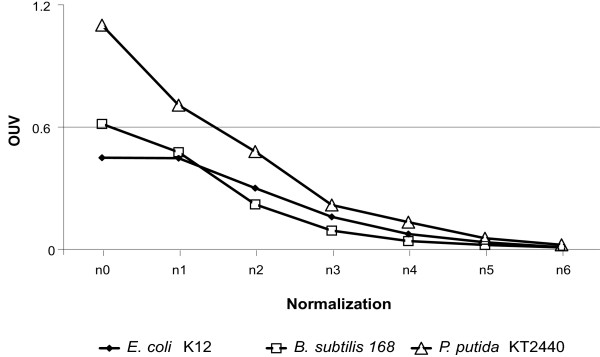
OUV of different heptanucleotide usage patterns from n0_7 mer to n6_7 mer determined for complete bacterial genomes.

OUV values of OU patterns from n0_7 mer to n6_7 mer were calculated for the complete genome sequences of *Bacillus subtilis *168, *Escherichia coli *K12 and *Pseudomonas putida *KT2440 (Fig. 1). OUV of n0_7 mer patterns depends strongly on GC-content getting minima in genomes with a GC content of about 50% such as in *E. coli *(Fig. 1) and maxima in AT-rich and, especially, GC-rich organisms, probably because OU is more strongly biased in GC-rich sequences [6,11]. Normalization of OU by mononucleotide frequency significantly removes this bias caused by GC-content (Fig. 1 and see ref. [6]). OUV n1_7 mer, however, is still high (Fig. 1). OUV decreases continuously with increase of the word length of internal normalization getting close to zero for n5 and n6 normalization of heptanucleotide usage (Fig. 1). This observation suggests that most OU constraints are caused by mononucleotide frequency and di-, tri- and tetranucleotide combinations while biases in frequencies of longer oligonucleotide words are probably just an extension of constraints of shorter component words.

### Local variations of OU patterns

**Figure 2:**
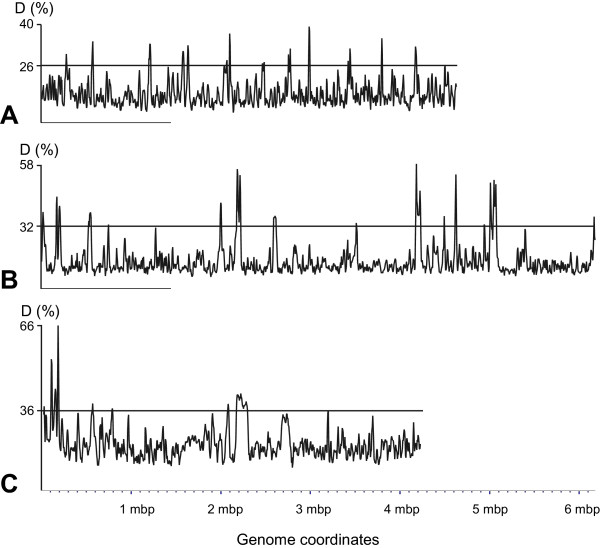
**Distances D between local n0_4 mer patterns and the global n0_4 mer patterns in the A)*E. coli *K12; B)*P. putida *KT2440 and C)*B. subtilis *168 chromosomes**. Local patterns were calculated for the sequence fragments of 8 kbp with sliding windows of 2 kbp. The 90% confidence interval of D values is depicted by horizontal lines. The loci with D-values exceeding the genomic confidence interval are considered as gene islands. The abscissa indicates the coordinates of the bacterial chromosomes as they were published in the NCBI database [27].

To analyze local variations of OU in bacterial genomes, the sliding window approach was used. 163 bacterial chromosomes of eubacteria and archaea published in the NCBI database were analyzed. Local OU patterns were calculated for 8 kb genome fragments with 2 kb sliding windows [6]. Fig. 2 shows the distances D of local n0_4 mer patterns in three selected bacterial genomes: *E. coli *K12, *P. putida *KT2440 and *B. subtilis *168 chromosomes. Genomic regions termed the 'core sequences' were characterized by OU patterns being similar to the global pattern of the chromosome. However, multiple genomic loci with alternative OU patterns that can make up more than 10% of the whole genome [11] were also detected in the three tested bacterial genomes (Fig. 2). Locally deviant OU patterns were found to comprise of heterogeneous subsets of parasitic and recent foreign DNA, ancient genes for ribosomal constituents (RNAs and proteins), multidomain genes and non-coding sequences with multiple tandem repeats.

**Figure 3:**
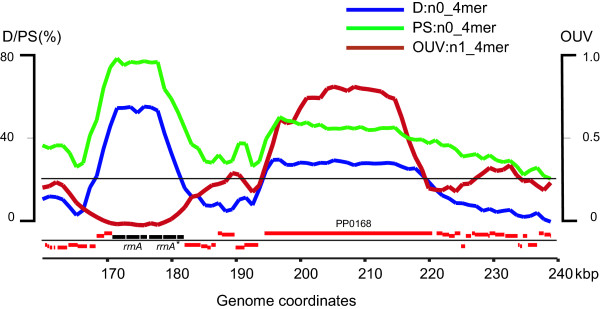
Curves of D:n0_4 mer, PS:n0_4 mer and OUV:n1_4 mer in a locus of the *P. putida *KT2440 genome covering two regions with atypical OU: rrnA-rrnA* gene cluster and a long multidomain gene PP0168 encoding the surface adhesion protein. Local OU patterns were analyzed in 5 kbp sliding windows with steps of 1 kbp. Curves are specified by a color code: blue for D, green for PS and brown for OUV. Protein coding genes are shown by red bars and genes for ribosomal RNAs are shown in black. The abscissa indicates the coordinates of the locus in the chromosome. The upper horizontal line shows the upper boundary of the 95% confidence interval of intragenomic deviation of D values. The lower horizontal line separates genes by their direction of transcription.

These functionally and evolutionarily unrelated subsets of atypical genomic loci were differentiated by the other OU statistical parameters: OUV and PS. These parameters often exhibited extreme values in detected atypical regions, however, their profiles were not congruent to each other. For example, consider the two adjacent gene islands in the *P. putida *KT2440 genome from 160 kbp to 240 kbp (Fig. 3). The first region (coordinates 170,815 – 180,000 bp) comprises of two tandem operons for ribosomal RNAs (*rrnA-rrnA'*) [12], while the second 26,045 bp sequence covers the largest *P. putida *gene PP0168 encoding the surface adhesion protein [11]. Both regions were recognized by alternative OU patterns (maximal D:n0_4 mer were 59% and 37.5%, respectively, see Figs. 2 and 3). Notably, OUV:n1_4 mer has its genomic minimum (0.08) in the first region but its genomic maximum (0.88) in the second region, whereas PS:n0_4 mer is maximal (74.7%) in the first region and it is closer to the average level (47.5%) in the second region. This example illustrates that the combination of several OU pattern parameters may be useful for the differentiation of unrelated gene subsets.

**Figure 4:**
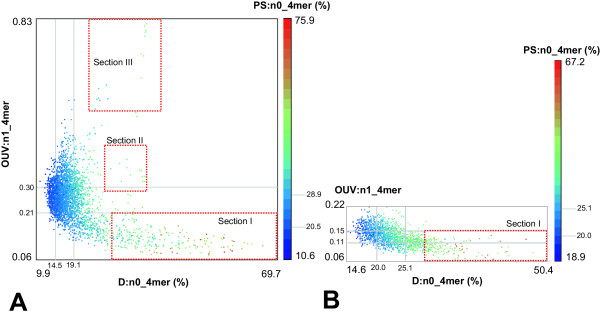
**Dot-plot presentation of 8 kb genomic fragments of A)*P. putida *KT2440 and B)*M. leprae *TN chromosomes**. Fragments of 8 kbp were generated with a sliding window 2 kbp. Each dot represents the D:n0_4 mer, OUV:n1_4 mer and PS:n0_4 mer values of one fragment. The latter parameter is depicted by a color code represented by the bar in the right part of the figure. The grey lines indicate borders of the inner quartiles of values for the corresponding OU statistical parameters.

The application of this procedure to a whole genome is shown in Fig. 4 for the cases of *P. putida *KT2440 and *Mycobacterium leprae *TN. Dots corresponding to the genome fragments were plotted in accordance with their D:n0_4 mer, OUV:n1_4 mer and PS:n0_4 mer values. The majority of fragments that represent the core genome clusters in one area. Three outlier groups detected in *P. putida *KT2440 and in the majority of other tested genomes were termed sections (Fig. 4A). Section I is heterogeneous and includes long intergenic regions, clusters of short hypothetical genes, laterally transferred elements and genes for ribosomal RNAs. The OU patterns of section I are characterized by low OUV and high PS. The operons for ribosomal RNAs exhibited the highest PS values (depicted by red dots, see Fig. 4). Genes for ribosomal proteins are localized in section II. This separation of ribosomal protein genes from the bulk genome was observed in most analyzed bacterial chromosomes but in some slow-growing microorganisms such as *M. leprae *these genes were not distinct from the core sequence (Fig. 4B). This observation is consistent with the notion that the codon usage in genes encoding ribosomal proteins is separate from the rest of genes in fast-growing bacteria but indistinguishable in slow-growing bacteria [13]. The differential codon usage of fast-growing bacteria has the consequence that ribosomal protein mRNA transcripts utilize other tRNA pools than the other mRNA species for the most abundant amino acids and hence the synthesis of the translational machinery is uncoupled from all other translational demands of the cell [14].

Section III encompasses the regions with outermost OUV (approximately 3 to 15 standard deviations of genomic OUV) and locus-specific OU patterns (large D values). The genetic repertoire covered by these loci is represented in Table 1. These regions typically comprise of one or more large multidomain genes of over 4 kbp in length or non-coding sequences with multiple tandem repeats. Examples are genes coding for surface proteins (*P. putida *KT2440, *Staphylococcus aureus *N315, *Xylella fastidiosa *Temecula 1), hemagglutinins and hemolysins (*Acinetobacter *sp., *Bordetella bronchiseptica *RB50, *Pseudomonas aeruginosa *PA01, *Pseudomonas syringae *DC3000, *X. fastidiosa *Temecula 1 and *Yersinia pestis *KIM), fatty-acid synthetases (*Corynebacterium efficiens *YS-314) and genes for proteins with an overrepresentation of a few amino acids (*Mycobacterium tuberculosis *H37Rv, *Streptomyces coelicolor *A3(2)). Many bacterial chromosomes lack these genetic elements. It seems that these genes or mulidomain regions are species specific. For example, consider the *M. leprae *genome lacking such genetic elements (Fig. 4B) in comparison with the closely related *M. tuberculosis *H37Rv (Table 1). The genetic elements of section III were not observed in the following tested genomes: *Aeropyrum pernix *K1, *Agrobacterium tumefaciens *C58, *Aquifex aeolicus *VF5, *Archaeglobus fulgidus *DSM4304, *Azoarcus *sp. EbN1, *Bacillus anthracis *Ames, *B. subtilis *168, *Bdellovibrio bacteriovorus *HD100, *Borrelia burgdorferi *B31, *Campylobacter jejuni *NCTC 11168, *E. coli *K12, *Enterococcus faecalis *V583, *Francisella tularensis *Schu 4, *Haemophilus influenzae *KW20, *Halobacterium *sp. NRC1, *Helicobacter pylori *J99, *Lactococcus lactis *IL1403, *Mesorhizobium loti *MAFF303099, *Prochlorococcus marinus *CCMP1375, *Pyrococcus furiosus *DSM 3638, *Salmonella enterica *Ty2, *Shigella flexneri *2457T, *Streptococcus pneumoniae *R6, *S. pyogenes *MGAS8232, *Treponema pallidum *Nichols.

**Table 1:** Genetic repertoire of loci characterized by atypical tetranucleotide usage patterns and extreme OUV (section III in Fig. 4) identified in bacterial chromosomes

**Genome**	**Genes and the encoded protein**	Start*	**Length (bp)**	Δ_D_^†^	Δ_OUV_^‡^
*Acinetobacter *sp.	putative hemagglutinin/hemolysin-related protein	923,008	11,136	3.11	4.13
	non-coding multiple repeats TTTAGAAA	2,448,000	5.600	2.24	17.33
*Bordetella bronchiseptica *RB50	BB1186: putative hemolysin	1,268,967	10,041	5.13	4.12
*Bradyrhizobium japonicum *USDA110	*blr325*: unknown	3,592,327	17,058	3.17	4.65
	*bll356*: unknown	3,930,196	10,326	6.23	5.02
	*bll371*: unknown	4,106,955	12,387	4.39	4.95
	*bll547*: unknown	6,017,600	12,633	5.04	6.16
*Corynebacterium efficiens *YS-314	*fasA*: fatty-acid synthase I	962,711	8,919	2.85	3.85
	*fasB*: fatty-acid synthase II	2,541,750	9,069	2.88	5.42
*Deinococcus radiodurans *R1 chromosome 1	DR1461-1462: hypothetical proteins	1,465,188	10,000	2.19	8.27
	non-coding tandem repeats CCCGCCC	519,833	8,415	7.06	8.42
*E. coli *O157:H7	Z0609, Z0615: RTX family exoproteins	581,356	20,160	1.82	9.43
*Mycobacterium tuberculosis *H37Rv	Rv0272c-Rv0279c hypothetical Gly-, Ala-rich proteins	328.573	10,499	1.52	9.15
	Rv0297-Rv0304c: hypothetical Gly-, Ala-, Asn-rich proteins	361,332	11,431	8.79	7.91
	Rv0355c: Asn-rich protein	424,775	9,903	8.31	10.91
	Rv0573c-Rv0578c: hypothetical Gly-rich proteins	665,849	10,066	0.60	4.72
	Rv0742-Rv0747: hypothetical Gly-rich proteins	832,979	7,876	1.24	3.97
	Rv1060-Rv1068c: hypothetical Gly-, Ala-rich proteins	1,183,506	8,641	1.04	5.54
	Rv1084-Rv1092c: hypothetical proteins	1,207,634	11.395	2.19	6.44
	multiple repeats CCGCCGCCA	1,630,636	7,592	2.33	8.84
	Rv2490c-Rv2494: hypothetical Gly-rich proteins	2,801,252	7,482	2.60	5.50
*Pseudomonas aeruginosa *PAO1	PA1874: hypothetical protein	2,036,441	7,407	2.61	5.61
*P. putida *KT2440	PP0168: Thr-rich surface adhesion protein	194,494	26,046	2.58	6.97
	PP0806: surface adhesion protein	926,690	18,930	1.17	4.39
*P. syringae *DC3000	PSPTO3229: filamentous hemagglutinin	3,629,677	18,825	2.34	7.87
*Rhodopirellula baltika *1	RB3077: putative cyclic nucleotide binding protein	1,588,083	18,024	1.62	6.19
	RB4375: large polymorphic membrane protein, probable extracellular nuclease;	2,242,933	9,171	3.23	7.09
	RB11769: probable aggregation factor core protein MAFp3	6,335,006	24,522	5.25	6.31
*Rhodopseudomonas palustris *CGA009	conserved hypothetical protein	1,459,664	9,891	2.61	3.38
	conserved hypothetical protein	1,475,303	13,008	2.89	4.18
*Sulfolobus solfataricus *P2	non-coding tandem repeats GAATTGAAAG	1,228,221	12,238	1.94	15.25
		1,253,000	5,000	1.50	8.67
		1,305,242	5,000	1.89	12.39
*Staphylococcus aureus *N315	*ebhA – ebhB*: large surface anchored proteins	1,437,928	20,142	4.04	10.07
	SA2447: similar to streptococcal hemagglutinin	2,755,253	6,816	3.03	9.29
*Streptomyces coelicolor *A3(2)	SC8F4.01c: Ala/Glu-rich protein	586,509	3.981	2.16	5.40
	SC2H4.02: hypothetical protein	6,836,057	6,552	2.86	4.80
*Xanthomonas campestris *ATCC33913	*yapH*: putative autotransporter adhesin	2,374,740	11,886	3.22	6.61
*Xylella fastidiosa *Temecula 1	non-coding sequence, multiple	1,183,606	11,095	1.31	9.81
	repeats (GGT)_n_	1,447,312	11,139	1.37	10.91
	*pspA1*: hemagglutinin	2,082,143	10,134	1.06	9.78
	*pspA2*: hemagglutinin	2,501,956	10,374	1.41	11.79
*Yersinia pestis *KIM	*irp1-2*: yersiniabactin peptide/polyketide synthetase;	2,654,642	15,867	4.27	6.05
	*yapH*: putative autotransporter adhesin	3,747,888	11,133	2.66	8.60
	y3579: putative filamentous hemagglutinin	3,961,333	9,888	3.31	4.32

* left coordinate of the locus in the chromosomal sequence;

^† ^deviation of the D:n0_4 mer value calculated for the locus from the mean genomic D:n0_4 mer in standard deviations;

^‡ ^deviation of the OUV:n1_4 mer value calculated for the locus from the mean genomic OUV:n1_4 mer in standard deviations;

Section I is heterogeneous. The genes for ribosomal RNAs are discerned from the other genes in section I by their extremely high PS of 60 – 70% that are usually the highest values in the genome. For further differentiation of the gene classes in section I, the next chapter describes the strategy to apply further OU statistical parameters to identify the subgroup of horizontally acquired elements.

### Identification of horizontally transferred elements

**Figure 5:**
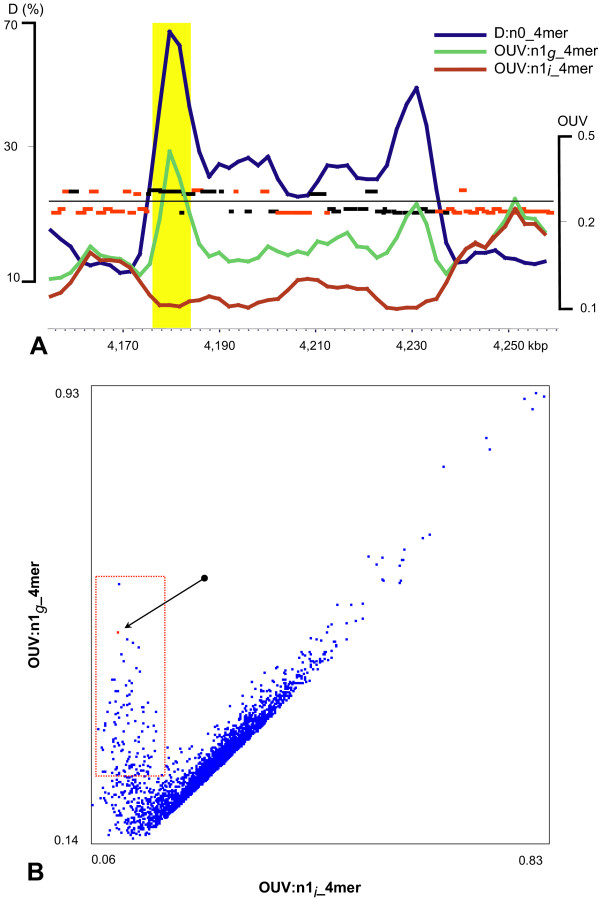
**Gene islands in the *P. putida *KT2440 genome identified by discordant OUV:n1_*i*__4 mer and OUV:n1_*g*__4 mer values A) in a local gene map and B) globally in the complete genome**. Genome fragments of 8 kbp were generated with a sliding window in step of 2 kbp. Red bars in figure A indicate protein coding genes and black bars-hypothetical genes. The horizontal line in the part A separates genes by direction of transcription. The yellow-shaded 8 kbp long fragment in A corresponds to the red dot indicated by an arrow in B.

Identification of laterally acquired elements in chromosomal sequences is of great importance because genomic islands often comprise pathogenicity and catabolic versatility determinants [15,16]. Two types of normalization of local OU patterns, – internal and global (see above), – were applied to visualize horizontally transferred gene islands within a genome sequence. The reason for introduction of these additional parameters was to improve the discrimination of foreign inserts in genome sequences. In core sequences, where the mononucleotide content is virtually the same as in the complete genome, results of internal and global normalization are identical in contrast to the laterally transferred loci characterized by an alternative mononucleotide content (in terms of GC-content, G/C-skew and A/T-skew). Correspondingly, values of OUV:n1_*i*__4 mer and OUV:n1_*g*__4 mer should merge in core sequences but widely diverge in gene islands (Fig. 5A). This concept was proven for genomes with known gene islands: SKIN element in *Bacillus subtilis *168 [17], phage related gene islands in *P. putida *KT2440 [11] and in *Salmonella enterica *Ty2 [18], pathogenicity island LEE in *E. coli *O157:H7 [19], IS-elements, pathogenicity and prophage islands in *Shigella flexneri *2457T [20], ISFtu1 element in *Francisella tularensis *Schu4 [21], *cag *pathogenicity island in *Helicobacter pylori *26695 [2] and 67 kbp gene island in *X. fastidiosa *9a5c [22]. All mentioned gene islands were successfully localized from the comparison of local with global OU patterns, however, no large foreign regions were observed in sequences of *Bradyrhizobium japonicum *and *Mesorhizobium loti *chromosomes, which both contain large symbiotic gene islands [23,24]. It looks as if these gene islands had been acquired a long time ago and hence their OU patterns adapted to the host genome OU signatures by genome amelioration [4,25].

An example for the identification of a laterally acquired gene island is shown in Fig. 5. The island in the chromosome of *P. putida *KT2440 has significantly divergent OUV:n1_*i*__4 mer and OUV:n1_*g*__4 mer values and D:n0_4 mer values beyond the 95% confidence interval of the complete chromosome (Fig. 5A). Since OUV:n1_*i*__*n*mer and OUV:n1_*g*__*n*mer in local patterns and the difference thereof are automatically calculated by the program, the method may be used for the high-throughput identification of horizontally transferred elements in bacterial genomes. Whereas OUV:n1_*i*__4 mer and OUV:n1_*g*__4 mer values are strongly correlated in the bulk *P. putida *genome, all islands show up by high OUV:n1_*g*__4 mer and low OUV:n1_*i*__4 mer values (Fig. 5B).

### Informative assignments of the OU statistical parameters

**Figure 6:**
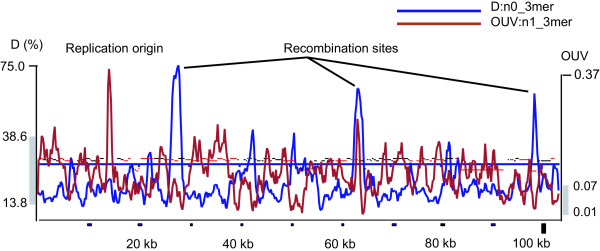
**Structural analysis of the complete sequence of the plasmid pKLC102 by local trinucleotide usage patterns**. Local OU patterns were analyzed in 1.2 kbp sliding windows with steps of 0.2 kbp. The scale indicates the coordinates of the plasmid sequence and separates genes by their direction of transcription. Red bars depict protein coding genes and black bars hypothetical genes. Grey bars along the D and OUV axes depict the 3-sigma ranges of fluctuation of D:n0_3 mer and OUV:n1_3 mer in a randomly generated sequence of the same length and mononucleotide contents as pKLC102.

The objective of our work was to analyze the informative assignment and applicability of different statistical parameters of OU. Di-, tri- and tetranucleotide usage patterns are charged with most information content (see Fig. 1). The optimal word length will provide maximal information about the question of interest. First, one has to consider the minimal sequence length that gives reliable OU statistics. The threshold values of the minimum length of sequence were calculated to be 0.3, 1.2, 5 and 20 kbp for di-, tri-tetra- and pentanucleotides, respectively [6]. However, to be informative, the window should of course be not too long, because otherwise short range fluctuations of OU will vanish. We recommend that the window should not be longer than 10-fold of its minimal length. Tetranucleotide (and, sometimes, pentanucleotide) usage patterns are more appropriate for the global analysis of sequences. A long sliding window silences signals from the local repeats and structural biases at the level of individual genes so that the characteristics of whole operons and gene islands become apparent. For a more detailed analysis of chromosomal loci or short genomes of bacterial plasmids and phages, tri- and dinucleotide usage patterns may be more appropriate. For example, in Fig. 6 the mosaic structure of the plasmid pKLC102 was recovered by investigation of local trinucleotide usage patterns (genomic fragments were segregated by 1.2 kbp sliding windows in steps of 200 bp). Three peaks of high D values depict recombination sites of the plasmid where additional genetic elements (transposons, integrons and gene cassettes) may be inserted [26]. A region with extremely high OUV:n1_3 mer corresponds to the putative replication origin of the plasmid [26].

To check whether the local fluctuations of OU parameters are statistically valid, a sequence of 100 kbp of mononucleotide content similar to pKLC102 was randomly generated. The ranges of 3-sigma fluctuation of D:n0_3 mer and OUV:n1_3 mer in the random sequence are depicted in Fig. 6 by vertical grey bars along the corresponding D and OUV axes. In the real sequences these values vary over a significantly larger range with the mean value of D smaller and the mean OUV higher than in the randomly generated sequence. (The plasmid pKLC102 sequence and the randomly generated sequence are included in the additional files as examples of source data files pKLC102.fts and random.fts, respectively.)

Normalization of OU by the internal component words changes the information assignment of OU biases. The three parameters D, PS and OUV were calculated for n0_4 mer, n1_4 mer, n2_4 mer and n3_4 mer local patterns for the pKLC102 genome and a part of the *E. coli *K12 chromosome from 1 Mbp to 2 Mbp. The former one is an example of a mosaic genome, and the latter one represents a regular bacterial chromosome. Correlation coefficients were calculated for respective OU statistical parameters determined for non-normalized and normalized local OU patterns. The correlation coefficients varied between 0.10 and 0.89 for pKLC102 and between 0.46 and 0.94 for *E. coli *(Table 2). This data demonstrates that n0, n1, n2 and n3 of 4 mer local patterns measure different characteristics of a sequence. In other words, the statistical parameters with different types of normalization provide non-redundant information that can be exploited for a refined analysis of genome organization. In case of tetranucleotide usage analysis four types of patterns exist: n0_4 mer, n1_4 mer, n2_4 mer and n3_4 mer. Each pattern type can be characterized by three parameters, D, PS and OUV that provide in total a comprehensive set of 12 non-redundant parameters for the nucleotide sequence analysis. Moreover, two subtypes of normalized OU patterns were introduced above, – with internal and global normalization, – that results in a total set of 21 non-redundant tetranucleotide usage statistical parameters each suitable for the refinement of functional gene classes in a raw nucleotide sequence.

**Table 2:** Correlation coefficients between D, PS and OUV of n0_4 mer local patterns with those of the corresponding n1, n2 and n3 normalized patterns

**Parameters**	**Normalization type**		
	**n1_4 mer**	**n2_4 mer**	**n3_4 mer**
plasmid pKLC102, window 5,000 bp, step 2,500 bp			
D:n0_4 mer	0.85*	0.82	0.40
PS:n0_4 mer	0.40	0.60	0.10
OUV:n0_4 mer	0.89	0.83	0.39
1 Mbp-2 Mbp locus of *E. coli *K12 chromosome, window 10,000 bp, step 5,000 bp			
D:n0_4 mer	0.94	0.84	0.63
PS:n0_4 mer	0.88	0.75	0.53
OUV:n0_4 mer	0.61	0.46	0.35

*Values in the cells of the table indicate the correlation coefficients between respective OU statistical parameters D, PS and OUV determined for n0 patterns and the normalized patterns n1, n2 and n3. For example, 0.85 is the correlation coefficient between series of values D:n0_4 mer and D:n1_4 mer determined for overlapping 5 kbp fragments of pKLC102.

## Conclusion

Bacterial genomes are not homogeneous but contain polymorphic blocks including horizontally transferred gene islands, non-coding sequences, long multidomain genes and ancient conserved gene clusters. The structural polymorphism of bacterial genomes may be effectively analyzed by local OU pattern signatures. A set of statistical approaches has been designed to perform this structural analysis of nucleotide sequences of bacterial genomes. These methods are useful for the visualization of regions with atypical oligonucleotide composition. The combination of the informative parameters that are 21 in case of tetranucleotide usage analysis, facilitates the prediction of gene classes. Moreover, many other subtypes of OU patterns may be additionally introduced. To this end, OU statistical analysis provides a valuable toolbox for the functional classification of regions and genes of interest prior to common-practice gene annotation.

A command line version of the Python program to apply the OU statistics methods mentioned above is available as additional file. To run the program, first the Python interpreted language program must be downloaded from the Web-site  and installed on the computer. The source DNA sequence (or sequences) should be saved in FASTA format in text file(s) with .FST file name extensions. Users may choose the OU statistical parameters to be calculated and the parameters of the sliding window by setting corresponding command line arguments. Many different OU parameters may be determined by a single run of the program and all FST files in the target folder will be processed continuously in a batch. For each source data file an output file in TXT format will be saved in the same folder. The full list of arguments and description of how to use the program are documented in the readme.doc file provided in the additional files. The program is fast enough to calculate all set of OU parameters mentioned in this paper for a complete bacterial genome of average length in 10–20 min depending on the computer performance.

Several general conclusions about OU in bacteria can be drawn from this report. First, most OU constraints are hidden in di-, tri- and tetranucleotide combinations that vanish with increasing word length (see Fig. 1). For example, in case of a hexamer the four possible heptamer words will have the same likelihood to occur next in the sequence. Hence, i)the analysis of the oligonucleotide distribution of up to 4-mers is sufficient to uncover all OU constraints in the sequence; and ii)neighbor effects are limited to dipeptides so that protein evolution is not skewed by oligonucleotide biases. Second, D and PS values are correlated in local patterns (see the examples for D:n0_4 mer and PS:n0_4 mer in Fig. 3 and 4). This observation is in accordance with the general trend in bacterial sequences to keep parity of frequencies of words and their reverse complements, in other words- a trend towards minimal PS [6]. OU parity is most pronounced for the OU pattern of the whole chromosome, whereas fluctuations of OU in local patterns lead to an increased PS. The exceptions are the laterally transferred elements with their island-specific OU signature. In this case, large D values of the local OU patterns may be associated with low PS (see blue and green dots in section I in Fig. 4).

## Methods

Sequences of 163 bacterial chromosomes including eubacterial and archaeal genomes published in the NCBI database [27] were analyzed in this study.

The OU statistical parameters-variance of word deviations (OUV); distances between patterns (D); pattern skew between leading and lagging strand (PS) were calculated by applying the algorithms described previously [6]. In a sequence of *L*_*seq *_nucleotides we calculated numbers of occurrence of overlapping *N*-long oligonucleotide words. There are 4^*N *^possible combinations of nucleotides and the total number of words in a sequence corresponds to the sequence length *L*_*seq*_. OU pattern was denoted as a matrix of deviations Δ[ξ1...ξN]
 MathType@MTEF@5@5@+=feaafiart1ev1aaatCvAUfKttLearuWrP9MDH5MBPbIqV92AaeXatLxBI9gBaebbnrfifHhDYfgasaacH8akY=wiFfYdH8Gipec8Eeeu0xXdbba9frFj0=OqFfea0dXdd9vqai=hGuQ8kuc9pgc9s8qqaq=dirpe0xb9q8qiLsFr0=vr0=vr0dc8meaabaqaciGacaGaaeqabaqabeGadaaakeaacqqHuoardaWgaaWcbaGaei4waSLaeqOVdG3aaSbaaWqaaiabigdaXaqabaWccqGGUaGlcqGGUaGlcqGGUaGlcqaH+oaEdaWgaaadbaGaemOta4eabeaaliabc2faDbqabaaaaa@3977@ of observed from expected counts for all possible words of the length *N*:

Δ[ξ1...ξN]=(C[ξ1...ξN]|obs−C[ξ1...ξN]|e)/C[ξ1...ξN]|0
 MathType@MTEF@5@5@+=feaafiart1ev1aaatCvAUfKttLearuWrP9MDH5MBPbIqV92AaeXatLxBI9gBaebbnrfifHhDYfgasaacH8akY=wiFfYdH8Gipec8Eeeu0xXdbba9frFj0=OqFfea0dXdd9vqai=hGuQ8kuc9pgc9s8qqaq=dirpe0xb9q8qiLsFr0=vr0=vr0dc8meaabaqaciGacaGaaeqabaqabeGadaaakeaacqqHuoardaWgaaWcbaGaei4waSLaeqOVdG3aaSbaaWqaaiabigdaXaqabaWccqGGUaGlcqGGUaGlcqGGUaGlcqaH+oaEdaWgaaadbaGaemOta4eabeaaliabc2faDbqabaGccqGH9aqpcqGGOaakcqWGdbWqdaWgaaWcbaGaei4waSLaeqOVdG3aaSbaaWqaaiabigdaXaqabaWccqGGUaGlcqGGUaGlcqGGUaGlcqaH+oaEdaWgaaadbaGaemOta4eabeaaliabc2faDjabcYha8jabd+gaVjabdkgaIjabdohaZbqabaGccqGHsislcqWGdbWqdaWgaaWcbaGaei4waSLaeqOVdG3aaSbaaWqaaiabigdaXaqabaWccqGGUaGlcqGGUaGlcqGGUaGlcqaH+oaEdaWgaaadbaGaemOta4eabeaaliabc2faDjabcYha8jabdwgaLbqabaGccqGGPaqkcqGGVaWlcqWGdbWqdaWgaaWcbaGaei4waSLaeqOVdG3aaSbaaWqaaiabigdaXaqabaWccqGGUaGlcqGGUaGlcqGGUaGlcqaH+oaEdaWgaaadbaGaemOta4eabeaaliabc2faDjabcYha8jabicdaWaqabaaaaa@6E5A@

where *ξ*_n _is any nucleotide A, T, G or C at the position 1, 2, 3, ... N in the N-long word; *C*_[*ξ*1...*ξN*]|*obs *_is the observed count of the word, [*ξ*_1_...*ξ*_*N*_]; *C*_[*ξ*1...*ξN*]|*e *_is the expected count and *C*_[*ξ*1...*ξN*]|0 _is a standard count estimated from the assumption of an equal distribution of words in the sequence: (C[ξ1...ξN]|0=Lseq×4−N
 MathType@MTEF@5@5@+=feaafiart1ev1aaatCvAUfKttLearuWrP9MDH5MBPbIqV92AaeXatLxBI9gBaebbnrfifHhDYfgasaacH8akY=wiFfYdH8Gipec8Eeeu0xXdbba9frFj0=OqFfea0dXdd9vqai=hGuQ8kuc9pgc9s8qqaq=dirpe0xb9q8qiLsFr0=vr0=vr0dc8meaabaqaciGacaGaaeqabaqabeGadaaakeaacqWGdbWqdaWgaaWcbaGaei4waSLaeqOVdG3aaSbaaWqaaiabigdaXaqabaWccqGGUaGlcqGGUaGlcqGGUaGlcqaH+oaEdaWgaaadbaGaemOta4eabeaaliabc2faDjabcYha8jabicdaWaqabaGccqGH9aqpcqWGmbatdaWgaaWcbaGaem4CamNaemyzauMaemyCaehabeaakiabgEna0kabisda0maaCaaaleqabaGaeyOeI0IaemOta4eaaaaa@476E@).

OU parameters of words of length *N *were normalized by shorter words *n *(0 ≤ *n *<*N*) as follows:

C[ξ1...ξN]|e=C[ξ1...ξN]|0
 MathType@MTEF@5@5@+=feaafiart1ev1aaatCvAUfKttLearuWrP9MDH5MBPbIqV92AaeXatLxBI9gBaebbnrfifHhDYfgasaacH8akY=wiFfYdH8Gipec8Eeeu0xXdbba9frFj0=OqFfea0dXdd9vqai=hGuQ8kuc9pgc9s8qqaq=dirpe0xb9q8qiLsFr0=vr0=vr0dc8meaabaqaciGacaGaaeqabaqabeGadaaakeaacqWGdbWqdaWgaaWcbaGaei4waSLaeqOVdG3aaSbaaWqaaiabigdaXaqabaWccqGGUaGlcqGGUaGlcqGGUaGlcqaH+oaEdaWgaaadbaGaemOta4eabeaaliabc2faDjabcYha8jabdwgaLbqabaGccqGH9aqpcqWGdbWqdaWgaaWcbaGaei4waSLaeqOVdG3aaSbaaWqaaiabigdaXaqabaWccqGGUaGlcqGGUaGlcqGGUaGlcqaH+oaEdaWgaaadbaGaemOta4eabeaaliabc2faDjabcYha8jabicdaWaqabaaaaa@4BE3@ if OU is not normalized, or C[ξ1...ξN]|e=C[ξ1...ξN]|n
 MathType@MTEF@5@5@+=feaafiart1ev1aaatCvAUfKttLearuWrP9MDH5MBPbIqV92AaeXatLxBI9gBaebbnrfifHhDYfgasaacH8akY=wiFfYdH8Gipec8Eeeu0xXdbba9frFj0=OqFfea0dXdd9vqai=hGuQ8kuc9pgc9s8qqaq=dirpe0xb9q8qiLsFr0=vr0=vr0dc8meaabaqaciGacaGaaeqabaqabeGadaaakeaacqWGdbWqdaWgaaWcbaGaei4waSLaeqOVdG3aaSbaaWqaaiabigdaXaqabaWccqGGUaGlcqGGUaGlcqGGUaGlcqaH+oaEdaWgaaadbaGaemOta4eabeaaliabc2faDjabcYha8jabdwgaLbqabaGccqGH9aqpcqWGdbWqdaWgaaWcbaGaei4waSLaeqOVdG3aaSbaaWqaaiabigdaXaqabaWccqGGUaGlcqGGUaGlcqGGUaGlcqaH+oaEdaWgaaadbaGaemOta4eabeaaliabc2faDjabcYha8jabd6gaUbqabaaaaa@4C5A@ if OU is normalized by empirical frequencies of all shorter words of the length *n*. The normalization was performed as follows. First at all, we calculated observed frequencies F[ξ1...ξn]
 MathType@MTEF@5@5@+=feaafiart1ev1aaatCvAUfKttLearuWrP9MDH5MBPbIqV92AaeXatLxBI9gBaebbnrfifHhDYfgasaacH8akY=wiFfYdH8Gipec8Eeeu0xXdbba9frFj0=OqFfea0dXdd9vqai=hGuQ8kuc9pgc9s8qqaq=dirpe0xb9q8qiLsFr0=vr0=vr0dc8meaabaqaciGacaGaaeqabaqabeGadaaakeaacqWGgbGrdaWgaaWcbaGaei4waSLaeqOVdG3aaSbaaWqaaiabigdaXaqabaWccqGGUaGlcqGGUaGlcqGGUaGlcqaH+oaEdaWgaaadbaGaemOBa4gabeaaliabc2faDbqabaaaaa@3966@ of *n*-long words in the sequence. Each word of length *N *can be represented as a consecutive set of *N *- *n *+ 1 overlapping component words of length *n*. For example, a pentamer ATGGC can be expressed as a set of 4 overlapping dimers: AT, TG, GG and GC. In a general case of a *N*-long word, a component word [*ξ*_1_...*ξ*_*n*_] reduces the set of available options for the next word in the sequence to 4 possible oligonucleotides: [*ξ*_2_...*ξ*_n_, A], [*ξ*_2_...*ξ*_n_, T], [*ξ*_2_...*ξ*_n_, G] and [*ξ*_2_...*ξ*_n_, C]. The relative frequencies of these words are:

F[ξ2...ξn,ξn+1]×[(F[ξ2...ξn,A]+F[ξ2...ξn,T]+F[ξ2...ξn,G]+F[ξ2...ξn,C])]−1
 MathType@MTEF@5@5@+=feaafiart1ev1aaatCvAUfKttLearuWrP9MDH5MBPbIqV92AaeXatLxBI9gBaebbnrfifHhDYfgasaacH8akY=wiFfYdH8Gipec8Eeeu0xXdbba9frFj0=OqFfea0dXdd9vqai=hGuQ8kuc9pgc9s8qqaq=dirpe0xb9q8qiLsFr0=vr0=vr0dc8meaabaqaciGacaGaaeqabaqabeGadaaakeaacqWGgbGrdaWgaaWcbaGaei4waSLaeqOVdG3aaSbaaWqaaiabikdaYaqabaWccqGGUaGlcqGGUaGlcqGGUaGlcqaH+oaEdaWgaaadbaWexLMBbXgBcf2CPn2qVrwzqf2zLnharyGvLjhzH5wyaGabaiaa=5gaaeqaaSGaeiilaWIaeqOVdG3aaSbaaWqaaiaa=5gacqGHRaWkcqaIXaqmaeqaaSGaeiyxa0fabeaakiabgEna0kabcUfaBjabcIcaOiabdAeagnaaBaaaleaacqGGBbWwcqaH+oaEdaWgaaadbaGaeGOmaidabeaaliabc6caUiabc6caUiabc6caUiabe67a4naaBaaameaacaWFUbaabeaaliabcYcaSiaa=feacqGGDbqxaeqaaOGaey4kaSIaemOray0aaSbaaSqaaiabcUfaBjabe67a4naaBaaameaacqaIYaGmaeqaaSGaeiOla4IaeiOla4IaeiOla4IaeqOVdG3aaSbaaWqaaiaa=5gaaeqaaSGaeiilaWIaa8hvaiabc2faDbqabaGccqGHRaWkcqWGgbGrdaWgaaWcbaGaei4waSLaeqOVdG3aaSbaaWqaaiabikdaYaqabaWccqGGUaGlcqGGUaGlcqGGUaGlcqaH+oaEdaWgaaadbaGaa8NBaaqabaWccqGGSaalcaWFhbGaeiyxa0fabeaakiabgUcaRiabdAeagnaaBaaaleaacqGGBbWwcqaH+oaEdaWgaaadbaGaeGOmaidabeaaliabc6caUiabc6caUiabc6caUiabe67a4naaBaaameaacaWFUbaabeaaliabcYcaSiaa=neacqGGDbqxaeqaaOGaeiykaKIaeiyxa01aaWbaaSqabeaacqGHsislcqaIXaqmaaaaaa@8BF7@

whereby the *F *values are the observed frequencies of the particular word of length *n *in the complete sequence and ξ is any nucleotide A, T, G or C. The expected count of a word [*ξ*_1_...*ξ*_*N*_] of length *N *in a *L*_*seq *_long sequence normalized by frequencies of *n*-mers (*n *<*N*) was calculated as follows:

C[ξ1...ξN]|n=Lseq×F[ξ1...ξn]×∏i=2N−n+1(F[ξi...ξi+n−2,ξi+n−1]∑XA,T,G,CF[ξi...ξi+n−2,X])
 MathType@MTEF@5@5@+=feaafiart1ev1aaatCvAUfKttLearuWrP9MDH5MBPbIqV92AaeXatLxBI9gBaebbnrfifHhDYfgasaacH8akY=wiFfYdH8Gipec8Eeeu0xXdbba9frFj0=OqFfea0dXdd9vqai=hGuQ8kuc9pgc9s8qqaq=dirpe0xb9q8qiLsFr0=vr0=vr0dc8meaabaqaciGacaGaaeqabaqabeGadaaakeaacqWGdbWqdaWgaaWcbaGaei4waSLaeqOVdG3aaSbaaWqaaiabigdaXaqabaWccqGGUaGlcqGGUaGlcqGGUaGlcqaH+oaEdaWgaaadbaWexLMBbXgBcf2CPn2qVrwzqf2zLnharyGvLjhzH5wyaGabciaa=5eaaeqaaSGaeiyxa0LaeiiFaWNaemOBa4gabeaakiabg2da9iabdYeamnaaBaaaleaacqWGZbWCcqWGLbqzcqWGXbqCaeqaaOGaey41aqRaemOray0aaSbaaSqaaiabcUfaBjabe67a4naaBaaameaacqaIXaqmaeqaaSGaeiOla4IaeiOla4IaeiOla4IaeqOVdG3aaSbaaWqaaiaa=5gaaeqaaSGaeiyxa0fabeaakiabgEna0oaarahabaWaaeWaaeaadaWcaaqaaiabdAeagnaaBaaaleaacqGGBbWwcqaH+oaEdaWgaaadbaGaemyAaKgabeaaliabc6caUiabc6caUiabc6caUiabe67a4naaBaaameaacqWGPbqAcqGHRaWkcqWGUbGBcqGHsislcqaIYaGmaeqaaSGaeiilaWIaeqOVdG3aaSbaaWqaaiabdMgaPjabgUcaRiabd6gaUjabgkHiTiabigdaXaqabaWccqGGDbqxaeqaaaGcbaWaaabCaeaacqWGgbGrdaWgaaWcbaGaei4waSLaeqOVdG3aaSbaaWqaaiabdMgaPbqabaWccqGGUaGlcqGGUaGlcqGGUaGlcqaH+oaEdaWgaaadbaGaemyAaKMaey4kaSIaemOBa4MaeyOeI0IaeGOmaidabeaaliabcYcaSiabdIfayjabc2faDbqabaaabaGaemiwaGfabaGaemyqaeKaeiilaWIaemivaqLaeiilaWIaem4raCKaeiilaWIaem4qameaniabggHiLdaaaaGccaGLOaGaayzkaaaaleaacqWGPbqAcqGH9aqpcqaIYaGmaeaacqWGobGtcqGHsislcqWGUbGBcqGHRaWkcqaIXaqma0Gaey4dIunaaaa@A127@

For further processing of OU statistics, the words were sorted by their Δ_[*ξ*1...*ξN*] _and the ranks of words instead the real values of deviations of observed from expected counts were used. The rank values (from 1 to 256 in the case of tetranucleotide analysis) were assigned to the words in accordance with their Δ[ξ1...ξN]
 MathType@MTEF@5@5@+=feaafiart1ev1aaatCvAUfKttLearuWrP9MDH5MBPbIqV92AaeXatLxBI9gBaebbnrfifHhDYfgasaacH8akY=wiFfYdH8Gipec8Eeeu0xXdbba9frFj0=OqFfea0dXdd9vqai=hGuQ8kuc9pgc9s8qqaq=dirpe0xb9q8qiLsFr0=vr0=vr0dc8meaabaqaciGacaGaaeqabaqabeGadaaakeaacqqHuoardaWgaaWcbaGaei4waSLaeqOVdG3aaSbaaWqaaiabigdaXaqabaWccqGGUaGlcqGGUaGlcqGGUaGlcqaH+oaEdaWgaaadbaGaemOta4eabeaaliabc2faDbqabaaaaa@3977@ values by ordering the words from the most overrepresented one (the greatest Δ[ξ1...ξN]
 MathType@MTEF@5@5@+=feaafiart1ev1aaatCvAUfKttLearuWrP9MDH5MBPbIqV92AaeXatLxBI9gBaebbnrfifHhDYfgasaacH8akY=wiFfYdH8Gipec8Eeeu0xXdbba9frFj0=OqFfea0dXdd9vqai=hGuQ8kuc9pgc9s8qqaq=dirpe0xb9q8qiLsFr0=vr0=vr0dc8meaabaqaciGacaGaaeqabaqabeGadaaakeaacqqHuoardaWgaaWcbaGaei4waSLaeqOVdG3aaSbaaWqaaiabigdaXaqabaWccqGGUaGlcqGGUaGlcqGGUaGlcqaH+oaEdaWgaaadbaGaemOta4eabeaaliabc2faDbqabaaaaa@3977@ to the least represented one (the lowest Δ[ξ1...ξN]
 MathType@MTEF@5@5@+=feaafiart1ev1aaatCvAUfKttLearuWrP9MDH5MBPbIqV92AaeXatLxBI9gBaebbnrfifHhDYfgasaacH8akY=wiFfYdH8Gipec8Eeeu0xXdbba9frFj0=OqFfea0dXdd9vqai=hGuQ8kuc9pgc9s8qqaq=dirpe0xb9q8qiLsFr0=vr0=vr0dc8meaabaqaciGacaGaaeqabaqabeGadaaakeaacqqHuoardaWgaaWcbaGaei4waSLaeqOVdG3aaSbaaWqaaiabigdaXaqabaWccqGGUaGlcqGGUaGlcqGGUaGlcqaH+oaEdaWgaaadbaGaemOta4eabeaaliabc2faDbqabaaaaa@3977@. This approach made the OU statistical parameters free from any dependence on the sequence length, provided that the sequence has a minimum length *L*_*min *_so that in a random sequence of the same length *L*_*min *_95% of all words of length *N *occur at least ten times (see above and [6]). Hence, local OU patterns that meet these requirements could be compared with the global pattern.

The distance *D *between two patterns was calculated as the sum of absolute distances between ranks of identical words (*w*, in a total 4^*N *^different words) in patterns *i *and *j *as follows:

D(%)=100×∑w4N|rankw,i−rankw,j|−Dmin⁡Dmax⁡−Dmin⁡
 MathType@MTEF@5@5@+=feaafiart1ev1aaatCvAUfKttLearuWrP9MDH5MBPbIqV92AaeXatLxBI9gBaebbnrfifHhDYfgasaacH8akY=wiFfYdH8Gipec8Eeeu0xXdbba9frFj0=OqFfea0dXdd9vqai=hGuQ8kuc9pgc9s8qqaq=dirpe0xb9q8qiLsFr0=vr0=vr0dc8meaabaqaciGacaGaaeqabaqabeGadaaakeaacqWGebarcqGGOaakcqGGLaqjcqGGPaqkcqGH9aqpcqaIXaqmcqaIWaamcqaIWaamcqGHxdaTdaWcaaqaamaaqahabaWaaqWaaeaacqWGYbGCcqWGHbqycqWGUbGBcqWGRbWAdaWgaaWcbaGaem4DaCNaeiilaWIaemyAaKgabeaakiabgkHiTiabdkhaYjabdggaHjabd6gaUjabdUgaRnaaBaaaleaacqWG3bWDcqGGSaalcqWGQbGAaeqaaaGccaGLhWUaayjcSdaaleaacqWG3bWDaeaacqaI0aandaahaaadbeqaaiabd6eaobaaa0GaeyyeIuoakiabgkHiTiabdseaenaaBaaaleaacyGGTbqBcqGGPbqAcqGGUbGBaeqaaaGcbaGaemiraq0aaSbaaSqaaiGbc2gaTjabcggaHjabcIha4bqabaGccqGHsislcqWGebardaWgaaWcbaGagiyBa0MaeiyAaKMaeiOBa4gabeaaaaaaaa@6530@

PS is a particular case of D where patterns *i *and *j *were calculated for the same DNA but for direct and reversed strands, respectively. D_max _= 4^*N*^(4^*N *^- 1)/2 and D_min _= 0 when calculating a D, or, in a case of PS calculation, D_min _= 4^*N *^if *N *is an odd number or D_min _= 4^*N *^- 2^*N *^if *N *is an even number [6].

The definition of OUV was provided in our previous paper [6].

The random sequence was generated by a in-house program using the Python randomizer [28].

## List of abbreviations

OU – oligonucleotide usage;

OUV – oligonucleotide usage variance;

PS – pattern skew;

D – distance between two OU patterns of an identical type.

## Authors' contributions

ONR did Python programming. Both authors contributed equally to all other presented data.

## Supplementary Material

Additional File 1 There is an additional ZIP archive file OligoWords for BMC Bioinf.zip comprising following documents: **OligoWords1.1.exe.py **- a command line version of the program implemented in Python2.2 [28]. **readme.doc **- description of the project in Word97 format. **pKLC102.fst**- sequence of the plasmid pKLC102 [26] in FASTA format that may be used as a source data file for the program OligoWords1.1.exe.py (see readme.doc). **random.fst **- a randomly generated sequence comparable with one of the plasmid pKLC102 by length and mononucleotide content. The file is in FASTA format that may be used as a source data file for the program OligoWords1.1.exe.py (see readme.doc). Click here for file
