# Supplementary material for: Differentiation of regions with atypical oligonucleotide composition in bacterial genomes
Source: BMC Bioinformatics. 2005 Oct 14;6:251. doi: 10.1186/1471-2105-6-251 (PMC1274298; doi:10.1186/1471-2105-6-251)
Supplement: Additional File 1 — There is an additional ZIP archive file OligoWords for BMC Bioinf.zip comprising following documents: OligoWords1.1.exe.py - a command line version of the program implemented in Python2.2 [28]. readme.doc - description of the project in Word97 format. pKLC102.fst- sequence of the plasmid pKLC102 [26] in FASTA format that may be used as a source data file for the program OligoWords1.1.exe.py (see readme.doc). random.fst - a randomly generated sequence comparable with one of the plasmid pKLC102 by length and mononucleotide content. The file is in FASTA format that may be used as a source data file for the program OligoWords1.1.exe.py (see readme.doc). [file 1471-2105-6-251-S1.zip › readme.doc]

The program may be executed on the computer where the Python interpreted language program is installed. The Python may be freely downloaded from the Web-site http://www.python.org/download/. The source DNA sequence (or sequences) should be saved in FASTA format in text file(s) with .FST file name extensions. For example:

>pKLC102

TGGTGGGTCGTGTAGGATTCGAACCTACGACCAATTGGTTAAAAGACAATTGGCCTA… (sequence in a single line or in multiple lines)

Users may choose the OU statistical parameters to be calculated and the parameters of the sliding window by setting corresponding command line arguments. Many different OU parameters may be determined by a single run of the program and all FST files in the target folder will be processed continuously in a batch. For each source data file an output file in TXT format will be saved in the same folder.

To run the program, select Start->Run. Enter the path to the **OligoWords1.1.exe.py** executable file and the list of argument in the command line as follows:

**C:\Python\OligoWords\OligoWords1.1.exe.py** task=n0_4mer:D;n0_4mer:PS;n1_4mer:V frame=5000 step=2500 path=.\data\ file=outfile

or arguments with commas:

**C:\Python\OligoWords\OligoWords1.1.exe.py** task=n0_4mer:D;n0_4mer:PS;n1_4mer:V, frame=5000, step=2500, path=.\data\, file=outfile

The order of the arguments does not matter but there should not be any spaces around ‘=’, ‘:’ and ‘;’. Some or all arguments may be omitted. In this case the default values of arguments will be used.

# Arguments

| **Argument** | **Options** | **Description** | **By default** |
| --- | --- | --- | --- |
| task= | n#_#mer,*task-n#* | First part is normalization from n0 to n6, but must be smaller than the word length; second part is word length from 2mer to 7mer; third part is the type of analysis (see below). *–n#* is optional pattern subtraction (see below). The list of tasks separated by ‘**;**’ may be submitted. In the output file the tasks will be organized in columns. | task= n0_4mer:D;n0_4mer:PS; n1_4mer:V |
|  | D | Distances between local oligonucleotide usage (OU) patterns and the standard pattern determined for the complete sequence. |  |
|  | GD | Globally normalized distances. Local patterns are normalized by frequencies of shorter constituent words determined for the complete sequence (in the mode D the frequencies of the component words are determined for every separate locus – the internal normalization).  This option makes sense only with normalized patterns (n1...n6). |  |
|  | PS | Pattern skew of local patterns |  |
|  | GPS | Globally normalized pattern skew. The same as PS, but OU patterns are normalized by frequencies of shorter constituent words determined for the complete sequence (in the mode PS the frequencies of the component words are determined for every separate locus – the internal normalization).  This option makes sense only with normalized patterns (n1...n6). |  |
|  | V | Oligonucleotide usage variance (OUV). |  |
|  | GV | Globally normalized OUV. OU patterns are normalized by frequencies of shorter constituent words determined for the complete sequence (in the mode V the frequencies of the component words are determined for every separate locus – the internal normalization).  This option makes sense only with normalized patterns (n1...n6). |  |
|  | GC | GC-content |  |
|  | GCS | GC-skew |  |
|  | ATS | AT-skew |  |
|  | -n# | This option returns the subtraction of values of patterns of different normalization types determined for the same loci. For example, the task:  **n0_4mer,PS-n1**  returns PS_n0 – PS_n1. Normalization by the mononucleotide content increase PS in coding sequences (negative values) and decrease PS in non-coding sequences with high mutation rate (positive values).  Subtraction may be applied for all tasks mentioned above. |  |
| frame= | None or an integer in bp | The length of the sliding window.  It can not be shorter than following:  7mer : 295000 bp,  6mer : 74000 bp,  5mer :18500 bp,  4mer : 4600 bp,  3mer : 1200 bp,  2mer :300 bp | 8 kbp |
| step= | None or an integer in bp | Can not be <= 0 or > frame  If None (by default), it is set to half of the frame length. | 2 kbp |
| path= | C:\path  .\path | Path to the active directory. By default the directory where the program was run is the active one. The program looks up for all source data files with extensions *.fst in the active directory and automatically processes all found files. | **.\** |
| file= | None or  *filename* without any extensions. | If None (by default), the output data will be saved in separate files with the same names as in source data files and extensions *.out. If a file with the same name exists in the active directory, it will be replaced.  If name of the file is provided, the output tables determined for all source data files found in the active directory will be saved in this file. The extension *.out will be added automatically. If a file with the same name exists in the active directory, the new data will be appended to the end of this file. | None |
| echo= | y  n | Echo mode  Switching the echo mode off increases speed of the program. | n |

If you want to change the default argument values, edit the lines 21-29:

```
        # arguments by default
        self.argv = {'task':'n0_4mer:D;n0_4mer:PS;n1_4mer:V;n1_4mer:GV',
                     'frame':8000,
                     'step':2000,
                     'path':'.',
                     'file':None,
                     'formate':'t',
                     'echo':'y'
                     }
```
